# Doping of carbon nanotubes by halogenated solvents

**DOI:** 10.1038/s41598-022-11162-3

**Published:** 2022-04-29

**Authors:** Patrycja Taborowska, Grzegorz Stando, Mika Sahlman, Maciej Krzywiecki, Mari Lundström, Dawid Janas

**Affiliations:** 1 Department of Organic Chemistry, Bioorganic Chemistry and Biotechnology, Silesian University of Technology, B. Krzywoustego 4, 44-100 Gliwice, Poland; 2 Hydrometallurgy and Corrosion, Department of Chemical and Metallurgical Engineering (CMET), School of Chemical Engineering, Aalto University, P.O. Box 16200, 00076 Aalto, Finland; 3 Institute of Physics-CSE, Silesian University of Technology, Konarskiego 22B, 44-100 Gliwice, Poland

**Keywords:** Carbon nanotubes and fullerenes, Electronic properties and materials, Design, synthesis and processing

## Abstract

Carbon nanotubes (CNTs) play a unique role in the area of flexible conductors as they have remarkably high electrical conductivity and bend easily without deformation. Consequently, CNTs are commonly deposited on substrates as conductive tracks/coatings. Halogenated solvents are often employed to facilitate the deposition process because they dry rapidly due to their high volatility. In this work, we report that halogenated solvents can dope CNTs considerably. The study showed that the use of dichloromethane, chloroform, or bromoform for the CNT deposition significantly impacts the chemical potential of the material, thereby modifying its charge transport characteristics. As a consequence, up to four-fold improvement in electrical conductivity is noted due to doping.

## Introduction

Carbon nanotubes (CNTs) draw considerable attention from the scientific community due to their extraordinary performance in almost every field. Electrical, optical, thermal, mechanical, and chemical properties of nanocarbon structures have motivated multiple efforts to develop methods of incorporating them into a macroscopic form such as films or fibers. Such macrostructures already have found numerous potential applications in modern electronics, starting from lightweight wires^1^, through sensors^2,3^, transistors^4–6^, to energy generators^7^ and supercapacitors^8^. Besides, the rapid development of photovoltaics^9,10^ and electrocatalysis^11,12^ also make SWCNTs highly applicable^13^.

Techniques of preparing CNT networks can be divided into dry and wet processing^14^, where the latter are mainly solution-based methods. A great variety of different solvents as well as stabilizers (surfactants^15^, polymers^16^, DNA^17^, etc.) are used in order to achieve homogeneous dispersion. Among them, organic solvents are often used without any additives and are thought to be removed from the film through evaporation without leaving any residues. However, more attention should be drawn to the selection of these solvents and their influence on the properties of the CNT macrostructures. If halogenated solvents affect CNTs in any way, this will impact several of the CNT film casting methods such as dip-coating^4,18^, spin-coating^19^, vacuum filtration^5,20^, or bar-coating^21^.

Halogen compounds such as dichloromethane, chloroform, and o-dichlorobenzene are widely used to coat substrates with CNTs. Chloroform is particularly popular for developing CNT films dedicated to field-effect transistors^1,4,5^ since a high evaporation rate is crucial for adaptation for mass production. Besides being less time–consuming due to its low boiling point, chloroform can also suppress possible agglomerations and aids in the alignment of the CNTs^6^. Furthermore, dichloromethane^20,22,23^ or o-dichlorobenzene (OCB)^18,19,24,25^ can also be used for similar CNT processing. The latter, in particular, is known for being able to form good dispersion of CNTs without aggregations or sediments. Consequently, many groups use it to prepare CNT networks.

However, as recently indicated by Kim et al., solvent selection may impact the electrical and thermoelectric properties of CNTs^21^. The authors noticed that using chloroform as a medium for CNT film formation increases the electrical conductivity and decreases the Seebeck coefficient of the conductive network. As a result, the Power Factor of the material was diminished four-fold at room temperature. A similar effect was reported by Moonoosawmy and Kruse, who observed that sonication of CNTs in solvents containing chlorine may dope the material due to the generation of Cl_2_ and HCl^26^. However, the literature lacks quantification of how different halogenated solvents impact the electrical/thermoelectric characteristics as well as the chemical composition of CNTs.

In this work, we thoroughly analyze this peculiarity by using single-walled carbon nanotubes (SWCNTs) and several halogenated solvents (dichloromethane, chloroform, and bromoform). We investigate how the values of electrical conductivity, Seebeck coefficient, and Power Factors of SWCNT films are affected as a function of halogen content and type as well as by the temperature. The goal was to probe the impact of these solvents on the carrier mobility/density of the SWCNT network. Moreover, we engage SEM, Raman spectroscopy and X-ray photoelectron spectroscopy to look for changes in microstructure, electronic characteristics and composition of the films, which could explain this behavior. Therefore, this paper aims to highlight the importance of the influence of said solvents, which appears overlooked.

## Materials and methods

### Assembly of SWCNT films using different solvents

**Figure 1:**
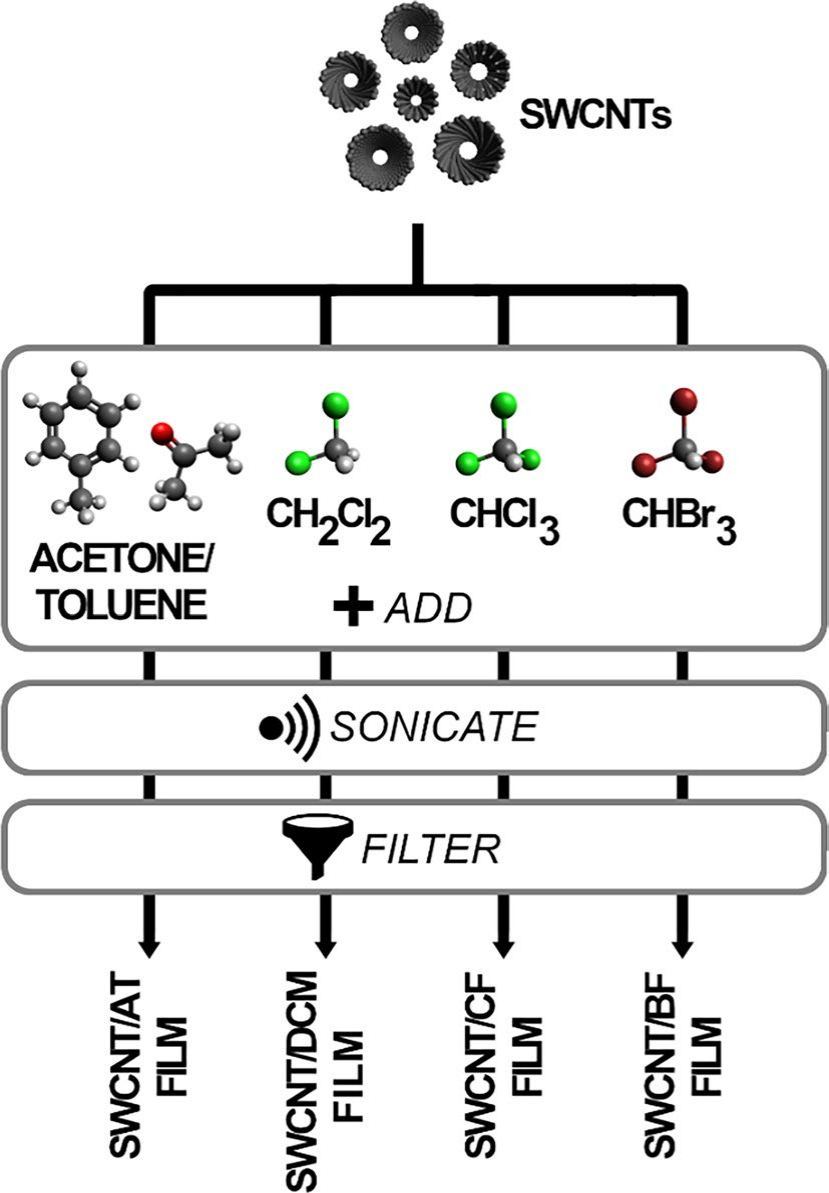
Preparation of SWCNT free-standing films using different solvents.

SWCNTs (Tuball™, OCSiAl), acetone (Avantor, Poland), toluene (Avantor, Poland), dichloromethane (Chempur, Poland), chloroform (Chempur, Poland), bromoform (Chempur, Poland) were all procured from commercial sources. All of the chemical compounds were of p.a. class. The free-standing SWCNT films were made using the previously described approach^27^ shown in Fig. 1.

In the preparation process, 60 mg of SWCNTs were introduced to 20 mL of either acetone/toluene mixture (1:1, w/w), dichloromethane, chloroform, or bromoform. Then, the mixture was sonicated at 100% amplitude for 15 min to obtain homogeneous SWCNT dispersion (UP200St sonicator, Hielscher, Germany) over an ice bath. The produced dispersion was then vacuum filtrated using PTFE membranes (pore size: 0.22 µm, diameter: 47 mm; Fisherbrand, Canada) and typical filtration apparatus consisting of Büchner funnel and a vacuum flask. Afterward, the films were left at the membrane under reduced pressure to facilitate evaporation of the solvents. Upon drying, the films easily detached from the substrate due to the low affinity of SWCNTs to Teflon. The obtained material was placed in a desiccator. Later, free-standing SWCNT films of ca. 100 µm in thickness were cut into 2 mm × 38 mm samples for characterization. The films are denoted as SWCNT/AT, SWCNT/DCM, SWCNT/CF, and SWCNT/BF for materials prepared in acetone/toluene mixture (AT), dichloromethane (DCM), chloroform (CF), and bromoform (BF), respectively.

### Characterization

Scanning Electron Microscope (SEM, JEOL JSM-7500FA, Japan) at the acceleration voltage of 15 kV was used to study the microstructure of the SWCNT films. Due to the high conductivity of the materials, they were not sputtered with gold.

Energy-dispersive X-ray spectroscopy (EDX) was engaged to probe the chemical composition of the material. The spectra were recorded from 0 to 20 keV using a Phenom ProX SEM with an online EDX detector coupled to the microscope. For each sample, three 25 µm × 25 µm areas were analyzed to obtain the average chemical composition of the sample.

Raman spectroscopy was employed to investigate the possibility of electronic and structural differences between the specimens. The inVia Renishaw Raman microscope with a laser wavelength of λ = 514 nm acquired data from 10 to 3200 cm^−1^. Spectra were obtained at several sample areas using extended acquisition time to ensure statistical significance and increase the signal-to-noise ratio, respectively.

X-ray photoelectron spectroscopy (XPS) and Ultraviolet photoelectron spectroscopy (UPS) were done using PREVAC EA15 hemispherical electron energy analyzer with 2D multi-channel plate detector. For XPS an Mg-Kα X-ray source (PREVAC dual-anode XR-40B source, excitation energy 1253.60 eV) was utilized to excite the sample. The system base pressure was equal to 9·10^−9^ Pa. Pass energy (PE) was set to 200 eV for survey scans (scanning step of 0.9 eV) and to 100 eV for particular energy regions (scanning step of 0.06 eV).

The UPS measurements were conducted with the same setup as for XPS. In this case, samples were irradiated with the He I spectral line (21.22 eV), and the PE was set to 5 eV. In order to assure the sample–analyzer work function separation, a bias voltage (− 5 V) was applied to the sample.

All XPS and UPS measurements were done with a normal take-off angle. The analyzer acceptance angle was limited to 15° together with the curved analyzer exit slit (0.8 × 25 mm) application for the highest energy resolution and unwanted energy dispersion limitation. The binding energy scale of the analyzer was calibrated first with respect to Au 4*f*7/2 (84.0 eV) region of the gold-covered sample placed at the same sample holder^28^.

The acquired spectra were fitted using CASA XPS® software. If not specified, the components were fitted with sum of Gauss (30%) and Lorentz (70%) functions while Shirley function was applied for the background subtraction.

The SWCNT films were attached with silver paint to a custom-made 4-point setup to evaluate electrical and thermoelectric properties. The external electrodes were placed on heating blocks and used to source current, while the internal electrodes were placed on temperature sensors and employed to measure voltage. The blocks were heated to temperatures from 30 to 105 °C, always keeping a 5 °C temperature difference between the sample ends during measurement. Pt100 resistance temperature detectors were used as temperature sensors. Generated voltage and internal resistance of the samples were measured using the quasi-steady state method with Keithley 2450 SMU and DMM6500 connected in a homemade setup. Source current used to measure resistance was 10 mA. All the devices were controlled with Raspberry Pi microcomputer and Python scripts. Other Python scripts were also employed to obtain the Seebeck coefficient, electrical conductivity, and Power Factor values. In order to calculate electrical conductivity, the thickness of the sample was measured 5 × each time with an electronic micrometer (LINEAR; UK). For every type of films, electrical conductivity values and Seebeck coefficients were determined for multiple samples, and the results were averaged.

## Results and discussion

**Figure 2:**
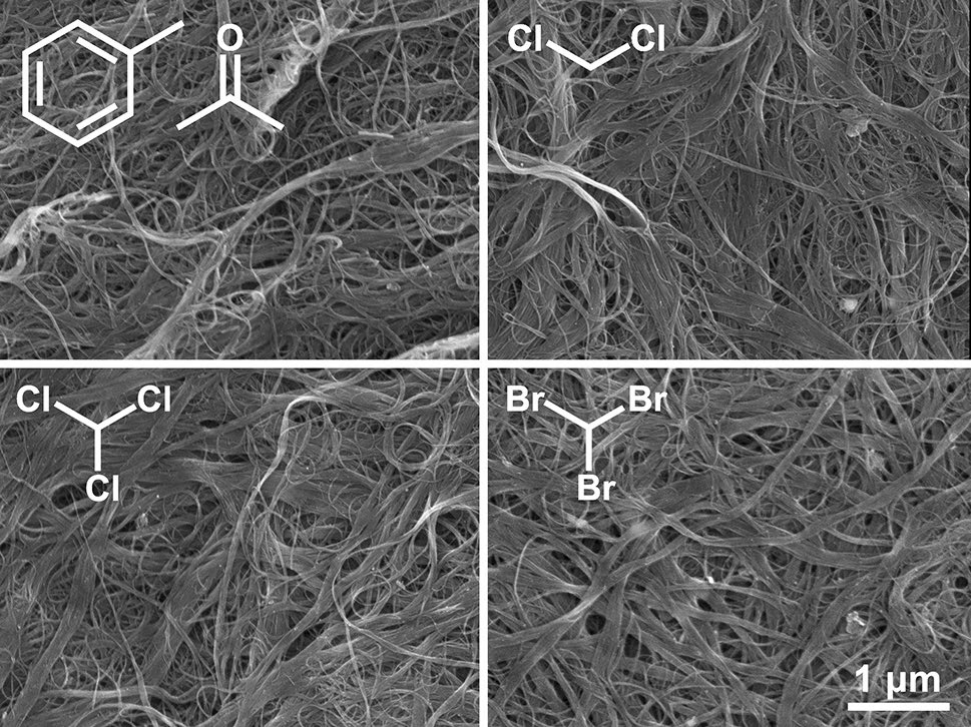
SEM micrographs of SWCNT free-standing films prepared using acetone/toluene mixture, dichloromethane, chloroform, and bromoform.

The study was initiated by characterizing the microstructure of the free-standing SWCNT films by SEM (Fig. 2). The micrographs revealed that the networks were of high purity regardless of the employed medium for the film formation. SWCNTs were well graphitized^29^, so the manufacturing method did not have a negative impact on the microstructure of the SWCNTs. In all cases, the amount of non-CNT impurities such as amorphous carbon on the surface was minimal, as in the work referred to above.

Furthermore, SWCNTs and SWCNT bundles were not arranged in any particular direction, as expected for a macroscopic network made by filtration. The size of voids separating SWCNTs appeared similar for all the formulations, suggesting no noticeable difference in the level of densification between the specimens. Consequently, purely from the geometrical perspective, the SWCNT films should have similar electrical conductivity as the average distance between SWCNTs and SWCNT bundles was essentially the same.

**Figure 3:**
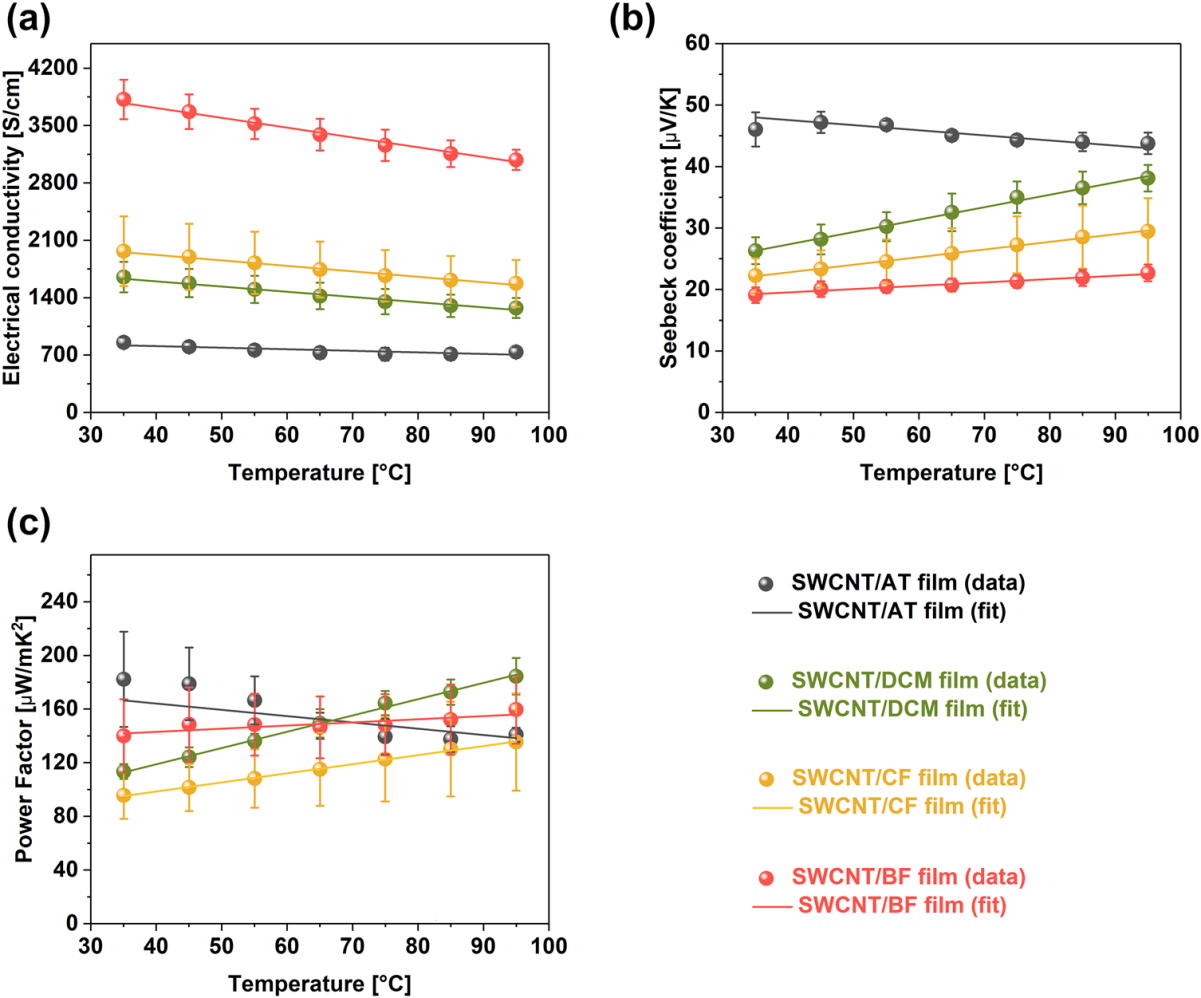
The impact of different liquid media for SWCNT film preparation on the values of (**a**) electrical conductivity, (**b**) Seebeck coefficients, and (**c**) power factors.

The electrical conductivity of films made in different solvents was measured. The room temperature conductivity was increased substantially when halogenated solvents were used as the medium (Fig. 3a). For example, SWCNT films prepared in acetone/toluene mixture showed the electrical conductivity of 853 ± 62 S/cm^30^, while it increased to 1652 ± 186 and 1966 ± 425 S/cm when dichloromethane and chloroform were used, respectively. An even more notable increase was observed for the SWCNT film specimen made in bromoform. Its electrical conductivity reached 3819 ± 241 S/cm, which was over a four-fold improvement compared to the material prepared in solvents not containing halogens. Considering the mild chemical nature of these compounds, the measured values are rather high. Kumanek et al. previously investigated how the dipping of SWCNT films in halogen-containing chemicals influences the electrical properties of the nanocarbon network^31^. The SWCNT films sonicated in the solvents mentioned above outperform most of the halides evaluated in this reference. This is a promising result as some of the chemicals tested before had a hazardous character or were difficult to handle. Concomitantly, this reveals that sonication of SWCNTs in the presence of halogens makes them much more potent doping agents.

Lastly, in all cases, as the temperature was increased, the electrical conductivity decreased, revealing that the SWCNT networks were of predominantly metallic character^32^. Due to this fact, the values of the Seebeck coefficients of differently prepared SWCNT films were satisfactory but not exceptional. The best thermoelectric performance is typically obtained for heavily-doped semiconductors, while the materials we analyzed were predominantly metallic. The room temperature Seebeck coefficient of the SWCNT/AT film reached 46 ± 3 μV/K, which matched the performance of other undoped thermogenerators based on SWCNTs (Fig. 3b)^7^. Conversely, the ensembles prepared in dichloromethane (SWCNT/DCM), chloroform (SWCNT/CF), and bromoform (SWCNT/BF) had Seebeck coefficients of 26 ± 2, 22 ± 3, and 19 ± 1 μV/K, respectively. Hence, the observed decrease in Seebeck coefficients strongly suggest that these routinely employed halogenated aromatic solvents for CNT processing may dope the material. Doping can increase the carrier concentration, which positively influences the electrical conductivity, but negatively affects the thermopower^33^, which is what we witnessed.

The combined impact of electrical conductivity (σ) and Seebeck coefficient (S) on the thermoelectric performance can be quantified by determining the Power Factor (PF) values, which are obtained as follows:

1 $$ {\text{PF}} =\upsigma {\text{S}}^{2} $$

The PFs for all the films are presented in Fig. 3c. It can be seen that the doping-induced enhancement of electrical conductivities almost fully compensated for the decrease in Seebeck coefficients. At the envisioned operational temperature for low-grade heat harvesting (300-400 K), the PF values were comparable, considering the established error bars. From the practical perspective, the PFs, which spanned from 141 ± 5 (SWCNT/AT) to 160 ± 25 μW/mK^2^ (SWCNT/BF), were rather competitive when compared with the state-of-the-art against other materials considered to contain only SWCNTs^7^.

**Figure 4:**
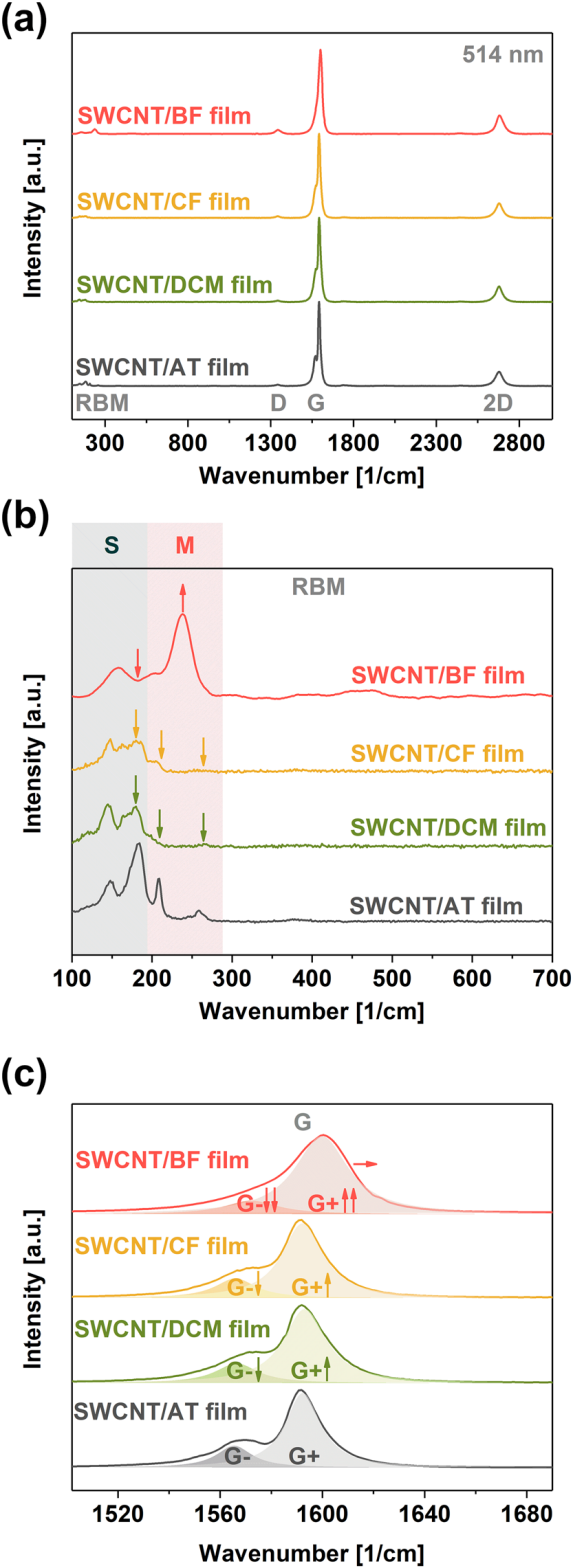
Analysis of SWCNT films prepared in different solvents by Raman spectroscopy (**a**) full spectra and magnifications of the (**b**) RBM (*S* semiconducting, *M* metallic) and (**c**) G peak areas.

Raman spectroscopy was employed to track the structural and electronic changes, causing the above-mentioned increase in the electrical conductivity (Fig. 4). It enables simple quantification of the disorder in nanocarbon, which can be measured by dividing the intensities of the defect-band D and the feature of graphitic vibrations G^30^.

The I_D_/I_G_ ratio obtained this way was very low for all the samples, thereby indicating that the SWCNT films were pristine and free of imperfections: 0.0164 ± 0.0012 (SWCNT/AC), 0.0149 ± 0.0014 (SWCNT/DCM), 0.0167 ± 0.0058 (SWCNT/CF), and 0.0488 ± 0.0013 (SWCNT/BF). We hypothesize that the slight increase in the I_D_/I_G_ ratios may be caused by chlorine and bromine radicals addition to the SWCNTs, as these reactive species may be generated by sonolysis of the halogenated solvents^34,35^. Maeda et al. showed that the sonication of SWCNTs in water might give oxygen-containing reactive species, which can functionalize the surface^36^. Analogously, it should be possible to form reactive halogen intermediates from the investigated halogen solvents. Bond energies for C–Cl and C–Br are 330 and 275 kJ/mol, respectively, while the bond energy of H–O is 464 kJ/mol. Thus, chemical modification of SWCNTs under sonication appears even more likely in halogenated solvents.

Furthermore, the generation of halogen moieties, which can dope SWCNT films, is most probable in the case of bromoform, which has the lowest electric dipole moment of 0.990 Debye, compared to 1.010 and 1.600 Debye for chloroform and dichloromethane, respectively. This partially explains why the enhancement of electrical conductivity is strongest for bromoform. To sum up, although the microstructure of the SWCNTs was not changed markedly, slight changes to the chemical composition of the material may have occurred, which could affect the system's resistance^37^. Other Raman spectra features were studied to elucidate this phenomenon further.

An intrinsic factor of resistance, which could influence the electrical conductivity of the networks, is the Fermi level of the SWCNT building blocks used to make such an ensemble. Changes to the Fermi level can be tracked by analyzing the Radial Breathing Mode area of the Raman spectra of SWCNTs (Fig. 4b). Peaks in this range come from particular SWCNTs, which resonate with the light of a specific wavelength illuminating the sample. Two populations can be discerned in this area: semiconducting and metallic^38^. It can be noticed that the use of halogenated solvents strongly suppressed the semiconducting features. Especially, the bromoform-doped SWCNT film experienced a substantial decrease in the intensity of semiconducting SWCNTs. This effect may be caused by the introduction of an impurity band near the Fermi level, which decreases the band gap for semiconducting SWCNTs^39^. Simultaneously, the peak of the metallic SWCNTs was considerably increased. Thus, the materials become more metallic upon doping with bromoform.

Furthermore, closer inspection of the G peak characteristics confirmed notable changes to the electronic properties of the SWCNTs (Fig. 4c). Firstly, the lineshape of this feature was modified by the presence of halogenated solvents. The intensity of the G- component decreased, and it started to blend with the G + feature because of changes in the electron–phonon coupling^26^. Moreover, another piece of evidence, which reveals significant changes to the material's electronic structure, comes from the analysis of the positions of the G + peak maxima^40^. While the shifts to this feature were not notable in the case of dichloromethane and chloroform, the application of bromoform shifted the position of the G + peak maximum by as much as 9 cm^−1^. This observation means that the material was powerfully p-doped by this solvent even though it was already p-doped by oxygen present in the ambient conditions^41^.

In light of the preceding observations, it can be concluded that the observed enhancement of electrical conductivity in halogenated solvents likely results from the change in chemical potentials of the SWCNT films. When different media are applied for their assembly, SWCNT films of various chemical potentials are formed, which strongly affects the electrical properties of the material. A similar effect was previously described for SWCNT films doped with halide compounds by simple immersion followed by drying^31^.

**Figure 5:**
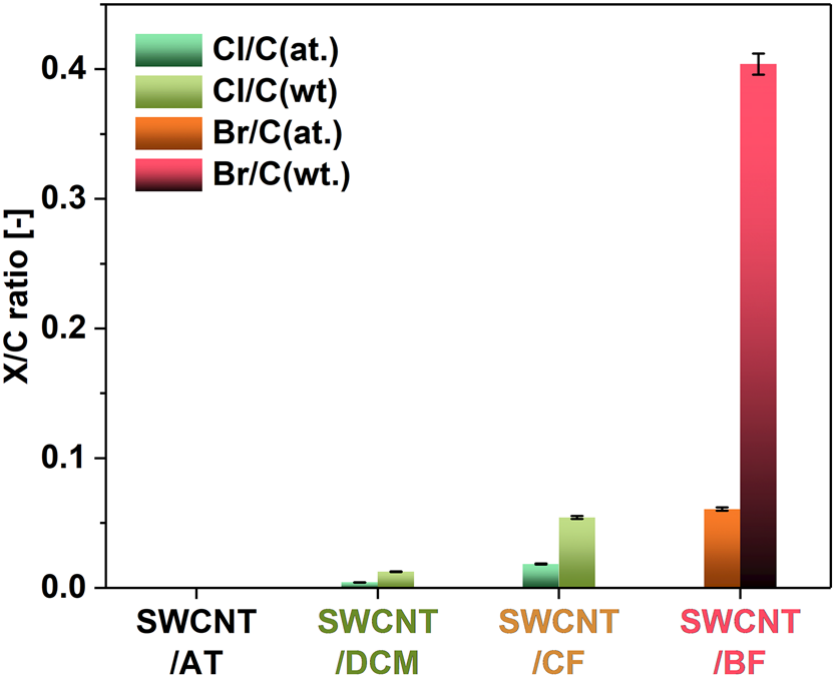
The atomic and weight ratios of halogens to carbon (X/C) in the SWCNT films prepared in the specified solvents determined by EDX.

To quantify the amount of halogens, which made a tangible change to the electrical conductivity of SWCNT films, EDX was employed (Fig. 5). The SWCNT film prepared in acetone/toluene mixture did not contain chlorine or bromine. However, the application of halogenated solvents had detectable amounts of these elements. For SWCNT/DCM and SWCNT/CF, the contents of chlorine were 0.004%at. and 0.012%wt. as well as 0.018%at. and 0.054%wt., respectively. Chloroform contains three chlorine atoms, which may explain why SWCNT/CF film exhibits a higher chlorine content, which translates to a more substantial enhancement of electrical conductivity (Fig. 3a). Regarding SWCNT/BF, the residual amount of bromine atoms was appreciable i.e. 0.061%at. and 0.404%wt. Bromine is heavier than chlorine, which justifies why its content by weight is so significant. We conclude that the halogen species generated by sonolysis (not the solvent molecules) interact strongly with the SWCNT sidewalls and affect the electronic characteristics even if they are present in relatively low amounts. Because bromoform has a rather high boiling point (150 °C^42^), compared with dichloromethane (40 °C^43^) and chloroform (61 °C^44^), one could argue that it is the residual solvent, which dopes the SWCNTs, especially in the case of this solvent and chloroform. However, the samples analyzed by EDX were dried in a desiccator for a week until reaching constant weight before the measurement. Furthermore, the EDX analysis was conducted inside of low-pressure environment of the SEM. It promotes the desorption of volatile species, so if the described effect was purely the result of solvent absorption, Cl_2_ or HCl^26^, it is unlikely that these compounds would be detected as these species have a relatively high vapor pressure.

**Figure 6:**
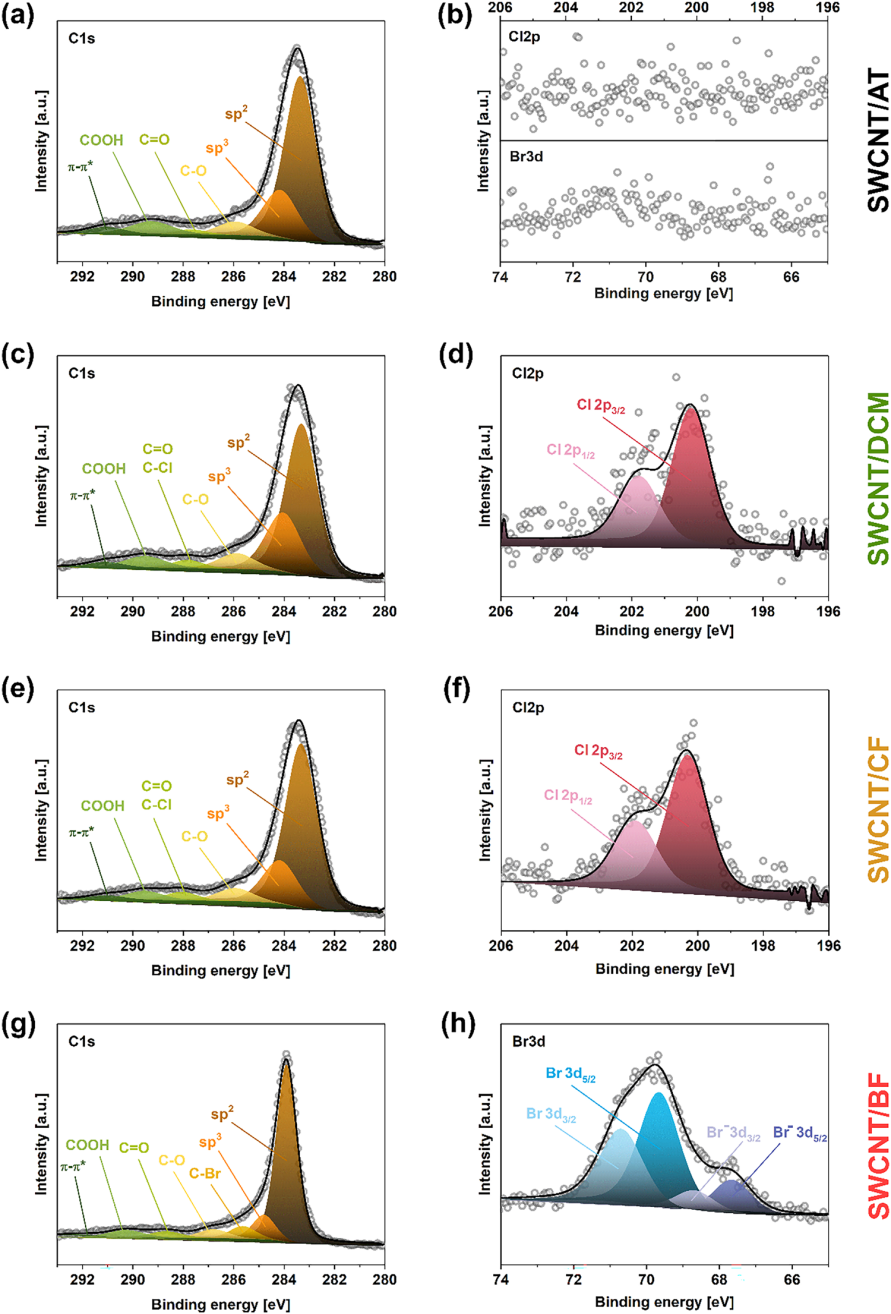
XPS spectra registered in (**a**, **c**, **e**, **g**) C1*s* and (**b**, **d**, **f**, **h**) Cl2*p*/Br3*d* areas for SWCNT/AT, SWCNT/DCM, SWCNT/CF, and SWCNT/BF samples.

**Figure 7:**
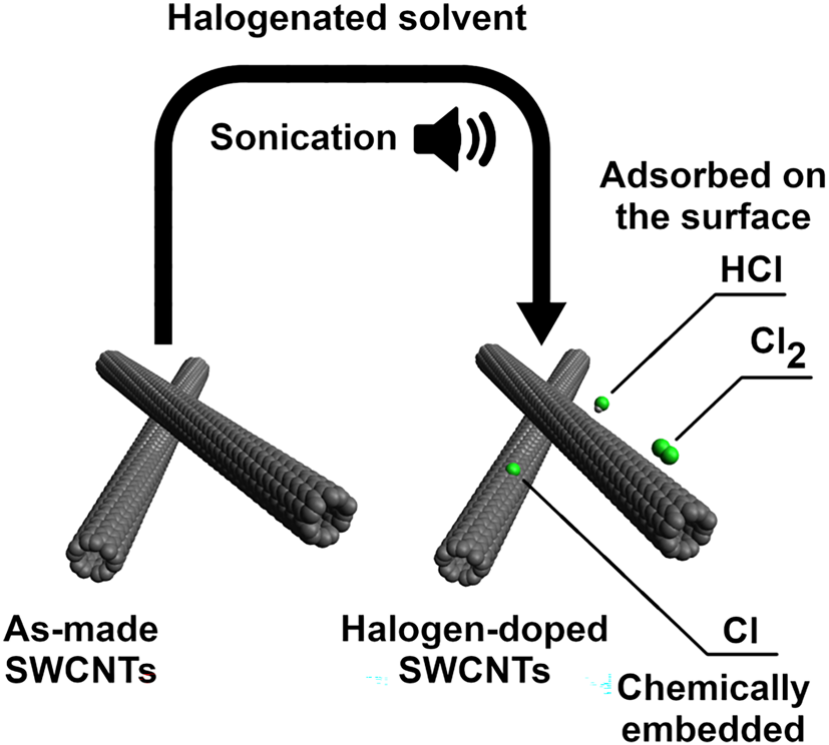
Modification of SWCNTs resulting from sonication in halogenated organic solvents. The above scheme represents the case of CH_2_Cl_2_ and CHCl_3_ solvents. Sonication of CHBr_3_ would analogously give HBr, Br_2_ and chemically-bound Br.

At this point, we focused on the investigation of this phenomenon by more sensitive XPS to unravel the bonding configuration present in the evaluated materials and investigate the possibility of chemical modification of SWCNTs (Fig. 6). The C1*s* spectra (left column) show that, regardless of the type of solvent used for SWCNT processing, the material is indeed of high crystallinity as in every case, the *sp*^2^ peaks have the highest intensity (Fig. 6a, c, e, g). Furthermore, for the SWCNT networks prepared in chlorine-containing solvents, the feature located at 288 eV that senses the presence of C=O and C–Cl bonds has noticeably increased intensity. Thus, handling SWCNTs in dichloromethane (Fig. 6c) and chloroform (Fig. 6e) leaves halides, which cannot be removed by prolonged evaporation. Similarly, when SWCNTs are handled in bromoform, the C–Br bond is clearly recognized at 285.7 eV. It has to be noted that the SWCNTs sonicated in halogen-free solvents do not contain chlorine or bromine atoms (Fig. 6b), so these elements are surely introduced by the processing. Therefore, besides simple adsorption of Cl_2_, HCl, Br_2_, and HBr on the SWCNT surface^26^, reactive chlorine or bromine radicals seem to attach to the SWCNT side-wall as well. A simplified hypothesized mechanism of the process is given below (Fig. 7).

Moreover, the Cl2*p* spectra of SWCNTs films produced in solvents containing chlorine (Fig. 6d, f) can be deconvoluted into two peaks: Cl 2*p*_3/2_ and Cl 2*p*_1/2_, which is indicative of spin–orbit coupling^45^. Interestingly, a doublet of doublets was registered Br3*d* spectrum for SWCNT film made in bromoform (Fig. 6h). The peaks at the low binding energy confirm the presence of bromine anions, which may form bromine chains inside of the SWCNT inner cavity^46^.

Finally, UPS was employed to quantify how the exposure of SWCNTs to halogen-containing solvents impacts the electronic characteristics of the material (Table 1). An apparent increase in electronic work function (WF) and the corresponding better energetic alignment of the Highest Occupied Molecular Orbital (HOMO) with respect to the Fermi energy (E_F_) denoted as E_F_ − E_HOMO_ were detected. These findings stayed in accordance with the electrical and thermoelectric results shown above. It was previously indicated that the highest increase in electrical conductivity resulted from the preparation of SWCNT films in bromoform: 3819 ± 241 S/cm versus 853 ± 62 S/cm for the SWCNT network manufactured from acetone/toluene medium. The SWCNT/BF sample had the largest Work Function (4.67 eV) and the lowest E_F_ − E_HOMO_ (2.24 eV), thereby explaining why it was most electrically conductive.

**Table 1:** Work function and E_F_ − E_HOMO_ determined for all SWCNT film samples.

Sample	Property	
	Work function (eV)	E_F_ − E_HOMO_ (eV)
SWCNT/AT	4.44	2.43
SWCNT/DCM	4.48	2.40
SWCNT/CF	4.55	2.30
SWCNT/BF	4.67	2.24

In summary, the results of this study show that even minute contents of halogens, supposedly generated by sonolysis, can strongly affect the capabilities of the SWCNT films to propagate electrical charge.

## Conclusions

The current study shows that the halogenated solvents commonly employed to prepare SWCNT dispersions in the forms of inks and paints can dope the material when the nanocarbon material is subjected to sonication in such media. Due to the presence of halogens, these solvents exhibit high volatility, which considerably shortens the drying time of the deposited SWCNT layers. As a consequence, they are widely employed in the field nowadays to create conducting tracks and coatings. However, this paper highlights that the solvents such as dichloromethane or chloroform (or the products of their sonolysis) can remain in the material and significantly affect the electronic nature of SWCNTs.

A considerable increase in their electrical conductivity was observed upon the combination of SWCNTs with the investigated halogen solvents under sonication. The most significant boost was noted in the case of bromoform, which increased the electrical conductivity of the SWCNT network from 853 ± 62 to 3819 ± 241 S/cm, compared with an SWCNT ensemble made in acetone/toluene mixture. Simultaneously, the Seebeck coefficient was reduced by more than a factor of two. These phenomena resulted from different chemical potentials of the SWCNT films, resulting from employing various solvents during their preparation. Furthermore, the ultrasound processing of SWCNTs in organic solvents appears to produce reactive halogen species, which may attack the SWCNT sidewall, thereby drastically changing the properties of the material.

## Data Availability

Available from the corresponding author upon a reasonable request.
